# Accurate diagnosis of lesions suspected of being caused by *Taenia solium* in body organs of pigs with naturally acquired porcine cysticercosis

**DOI:** 10.1371/journal.pntd.0007408

**Published:** 2019-06-25

**Authors:** Charles G. Gauci, Chrisostom Ayebazibwe, Zachary Nsadha, Chris Rutebarika, Ishab Poudel, Keshav Sah, Dinesh Kumar Singh, Andrew Stent, Angela Colston, Meritxell Donadeu, Marshall W. Lightowlers

**Affiliations:** 1 Faculty of Veterinary and Agricultural Sciences, University of Melbourne, Werribee Victoria, Australia; 2 National Animal Disease Diagnostics and Epidemiology Centre (NADDEC), Ministry of Agriculture Animal Industry and Fisheries, Entebbe, Uganda; 3 College of Veterinary Medicine, Animal Resources and Biosecurity, Makerere University, Kampala; 4 ANISOLUTIONS International Ltd, Entebbe, Uganda; 5 Heifer International, Kathmandu, Nepal; 6 Department of Pathology and Clinics (HOD), Tribhuvan University, Institute of Agriculture and Animal Science, Rampur Campus, Chitwan, Nepal; 7 GALVmed, Galana Plaza, Kilimani, Nairobi Kenya; 8 Initiative for Neglected Animal Diseases (INAND), Midrand, South Africa; IRNASA, CSIC, SPAIN

## Abstract

The definitive method for diagnosis of porcine cysticercosis is the detection of cysticerci at necropsy. Cysts are typically located in the striated muscle and brain. Until recently *Taenia solium* cysticerci have not been definitively identified in other tissue locations, despite several comprehensive investigations having been undertaken which included investigation of body organs other than muscle and brain. Recently a study conducted in Zambia reported 27% infection with *T*. *solium* in the liver of pigs with naturally acquired porcine cysticercosis, as well as some *T*. *solium* infection in the lungs and spleen of some animals. We investigated the cause of lesions in sites other than the muscle or brain in a total of 157 pigs from *T*. *solium* endemic regions of Uganda and Nepal which were subjected to extensive investigations at necropsy. Lesions which had the potential to be caused by *T*. *solium* were characterised by macroscopic and microscopic examination, histology as well as DNA characterisation by PCR-RFLP and sequencing. Lesions were confirmed as being caused by *Taenia hydatigena* (both viable and non-viable), by *T*. *asiatica* and *Echinococcus granulosus* (in Nepal) and nematode infections. No *T*. *solium*-related lesions or cysticerci were identified in any tissue other than muscle and brain. It is recommended that future evaluations of porcine cysticercosis in aberrant tissue locations include DNA analyses that take appropriate care to avoid the possibility of contamination of tissue specimens with DNA from a different tissue location or a different animal. The use of appropriate control samples to confirm the absence of cross-sample contamination is also recommended.

## Author summary

The only currently available method for accurate assessment of the prevalence of porcine cysticercosis is to undertake detailed examination of tissues at necropsy. Most investigations have found that in pigs, *Taenia solium* encysts in the striated muscles and nervous tissue. Recently an investigation of porcine cysticercosis carried out in Zambia described 27% of infected pigs as having *T*. *solium* cysts in the liver, as well some animals having cysts in other unusual tissues locations. We collected lesions from the tissues of pigs derived from *T*. *solium* endemic areas in Uganda and Nepal. Particular care was taken to avoid cross contamination of specimens with *T*. *solium* DNA from other tissue sites or from other animals. Control tissue samples were collected from the same organs as lesions were collected, but from sites where no lesion was present. The samples were assessed macroscopically, histologically and by PCR-RFLP analysis of DNA isolated from the lesions. Evidence was obtained for lesions in tissues other than the striated muscles and nervous tissue being caused by *Taenia hydatigena*, *Taenia asiatica*, *Echinococcus granulosus* and nematode parasites, however no evidence was found for the presence of any lesion in these tissues being caused by *T*. *solium*.

## Introduction

*Taenia solium* is a cestode parasite which is an important cause of human morbidity and mortality, particularly in many developing countries where sanitary conditions and pig rearing practices favour the parasite’s transmission. In endemic countries, *T*. *solium* has been found to be associated with 29% of cases of epilepsy [1]. Cysticercosis caused by *T*. *solium* is recognised by the World Health Organization as a Neglected Tropical Disease [2].

*T*. *solium* is a zoonotic parasite with pigs acting almost exclusively as the intermediate host responsible for transmission. Completion of the parasite’s life cycle has been prevented in many developed countries through improvements in sanitation and hygienic pig rearing practices, which prevent the animals from being exposed to human faeces. Similar improvements in developing countries where *T*. *solium* is currently endemic would be expected to reduce the incidence of human cysticercosis. However, large areas of Africa, Asia and Latin America where *T*. *solium* is endemic remain economically disadvantaged, presenting a substantial hindrance to implementation of control programs. New tools have become available for prevention or treatment of porcine cysticercosis [3], and the existence of these tools has encouraged an increasing number of efforts to evaluate different control strategies.

A critical aspect of implementing a control program for cysticercosis is the evaluation of the program’s effectiveness [4]. The most common tools used for monitoring *T*. *solium* control have been the evaluation of changes in the prevalence of human taeniasis and/or porcine cysticercosis. Monitoring changes in taeniasis is hampered by the tapeworm often occurring at low prevalence in the population, even in many highly endemic areas, and the common co-endemicity of other species of *Taenia* which cause false positive reactions in some tests for taeniasis [4]. Evaluation of changes in porcine cysticercosis has been the most commonly used method for monitoring the impact of *T*. *solium* control measures. The relatively high prevalence of porcine cysticercosis in endemic areas, the short life span of pigs and their critical, direct role in transmission of the parasite, all favour evaluation of changes in the incidence of porcine cysticercosis as the most practical method for evaluating *T*. *solium* control efforts at project level.

Serological methods for evaluation of the prevalence of porcine cysticercosis have been found to be highly non-specific [4]. For this reason, direct measures of infection are currently the only reliable method for diagnosis of porcine cysticercosis. Accurate diagnosis requires detailed post-mortem examinations involving the slicing of affected tissues to count cysts. In pigs, cysticerci occur most commonly in striated muscle tissues and brain.

Mature, viable *T*. *solium* cysticerci are readily identified macroscopically and viability can be confirmed simply by demonstration of evagination of excised cysticerci [5]. In natural and experimental infections, some or all cysts may die in the tissues. Necrotic lesions may contain a detectable, although non-viable cysticercus, the presence of which confirms *T*. *solium* cysticercosis. Non-viable lesions caused by *T*. *solium* which contain no sign of the parasite may also occur and some of these can be diagnosed using specific detection methods for parasite DNA.

Most studies of the tissue localization of *T*. *solium* in pigs have found the cysts to be restricted to the striated muscle tissue and nervous tissue. In rare circumstances of intense infection, cysts have been described also in the liver, lung and spleen [6]. Recently, Chembensofu et al. [7] found a high prevalence of *T*. *solium* cysticerci in naturally infected pigs in Zambia in tissues other than the striated muscle or nervous tissues. Chembensofu et al. found cysts in unusual tissue locations at a high frequency, even though other studies examined relatively large numbers of infected pigs and included very heavily infected animals and no cysts were found in unusual locations [8, 9]. Chembensofu et al. confirmed *T*. *solium* diagnosis by DNA analyses using PCR-restriction fragment length polymorphism (PCR-RFLP).

A field trial of the use of TSOL18 vaccination and medication for control of *T*. *solium* transmission by pigs was undertaken recently in the Banke district of Nepal [10]. Among 69 pigs examined at the conclusion of that trial, as well as cysts in striated muscle and nervous tissue, lesions were detected also in the liver and lungs of numerous animals. Similarly, we investigated the occurrence of cystic lesions in free-ranging pigs from *T*. *solium* endemic areas in Bukedea and Kumi Districts of Uganda and identified a number of animals containing suspect lesions in the liver and lungs that could possibly have been caused by *T*. *solium*. Here we detail the results of investigations that were undertaken which sought to identify the cause of these lesions in the Nepalese and Ugandan pigs, paying particular attention to the evaluation of control samples so as to provide evidence for the absence of false positive results in PCR-RFLP due to sample contamination.

## Methods

### Assessment of pig infections

Sixty-nine pigs of slaughter age and weight underwent extensive post mortem analyses in Nepal while 98 pigs were necropsied in Uganda. Post mortem methods are detailed by Poudel et al. [10]. The viscera were removed and the tongue, masticatory muscles, brain, heart, liver, lungs, both kidneys and the full diaphragm were retained in numbered containers. The muscles from each side of the carcass were dissected from the bones. All the retained organs, and muscles of the right-hand side of the carcass, were sliced by hand at intervals of approximately 3 mm and examined meticulously for the presence of *T*. *solium* cysticerci or other lesions. When no cysticerci were detected in the tongue, masticatory muscles, diaphragm, brain or muscles from the right-hand side of the carcass, the muscles of the left-hand side of the carcass were also sliced. Cysticerci in the striated muscles and brain were recorded and characterised as viable or non-viable [10]. For lesions identified in organs other than the striated muscle or brain, particularly in the liver and lung, representative samples were taken for DNA analyses, histological analyses and examination under a dissection microscope (Olympus model SF10). Specimens for histology were placed in 10% buffered formalin. Samples for DNA analyses were taken with care to minimize the potential for cross contamination of the samples with DNA from any other source. Samples were excised with a new, sterile scalpel blade and placed into a >10x excess volume of RNAlater tissue storage reagent (Sigma-Aldrich). Control tissue samples were taken from the same organ using a different, new scalpel blade, from sites adjacent to the place where the lesion had been sampled but which contained normal tissue only.

### DNA isolation from pig lesions

The same procedure was used for lesions and control tissue samples. They were washed with DNA extraction buffer (50 mM Tris pH 8.0, 50 mM EDTA, 100 mM NaCl, 0.5% SDS) to remove RNAlater storage buffer. DNA was extracted by digestion with Proteinase K (0.2 mg/ml, Promega) in DNA extraction buffer at 56°C for at least 16 hours or until proteinaceous material was completely dissolved. The DNA was purified by extraction with an equal volume mixture of phenol/chloroform and centrifugation at 18,000g for 15 min. The phenol/chloroform extraction was repeated in a new microfuge tube and residual phenol was removed by chloroform extraction and centrifugation. The DNA was precipitated by the addition of two volumes of absolute ethanol, incubation at 4°C for at least 1 h and centrifugation at 18,000g. DNA pellets were washed with 70% ethanol, centrifuged, dried after removal of ethanol and dissolved in sterile deionized water. The concentration of DNA in each sample was measured using a spectrophotometer (NanoDrop ND-1000, Thermo Scientific) and stored at -20°C.

For use in control PCR reactions, identical methods were used to isolate DNA from specimens of a variety of parasite species stored at -80°C.

### PCR analyses

DNA isolated from pig tissue lesions was amplified using primers described by Poon et al [11] as pan-nematode, having been designed so as to amplify DNA from a variety of nematode parasites. These primers targeted a 166 bp fragment of the COX1 gene: pan_nematode_cox1_692F 5′-TGTCTTTACCWGTTTTRGCTGG-3′ and pan_nematode_cox1_835R 5′-CCGAAAGCAGGYAAAATHARAA-3′. PCR conditions were the same as those described for the cestode PCR detailed below, except an annealing temperature of 50°C was used, with *Ascaris suum* and *Ascaris lumbricoides* DNA used as positive controls.

The mitochondrial 12S ribosomal RNA gene was amplified by PCR using the following primers originally described by Geysen et al. [12]: TaenF, 5’ GTTTGCCACCTCGATGTTGACT 3’ and ITMTnR, 5’ CTCAATAATAATCGAGGGTGACGG 3’. These primers were selected for PCR amplification due to the high conservation of DNA sequence at this locus, allowing species identification by targeting the 890 bp of the mitochondrial 12S ribosomal RNA gene. PCR amplification was based on the methods also described by Rodriguez-Hidalgo et al. [13] and Somers et al. [14] and was performed in a final volume of 25 μl with the following modifications: 1 μl template DNA (2 ng/ μl), 5 μl 5X SuperFi Buffer (Invitrogen, including 7.5 mM MgCl_2_), 0.5 μl (10 mM) dNTP mix, 1.25 μl (10 μM) of each forward and reverse primer, 0.25 μl (2 U/μl) Platinum SuperFi DNA Polymerase (Invitrogen), and 15.75 μl sterile de-ionized water. The PCR reactions were performed in a Bio-Rad T100 thermal cycler using the following conditions: initial denaturation (98°C, 30 s), followed by 35 cycles of denaturation (98°C, 10 s), annealing (63°C, 10 s) and extension (72°C, 90 s), and a final extension step (72°C, 5 min).

### PCR-restriction fragment length polymorphism (PCR-RFLP) analysis

**Fig 1:**
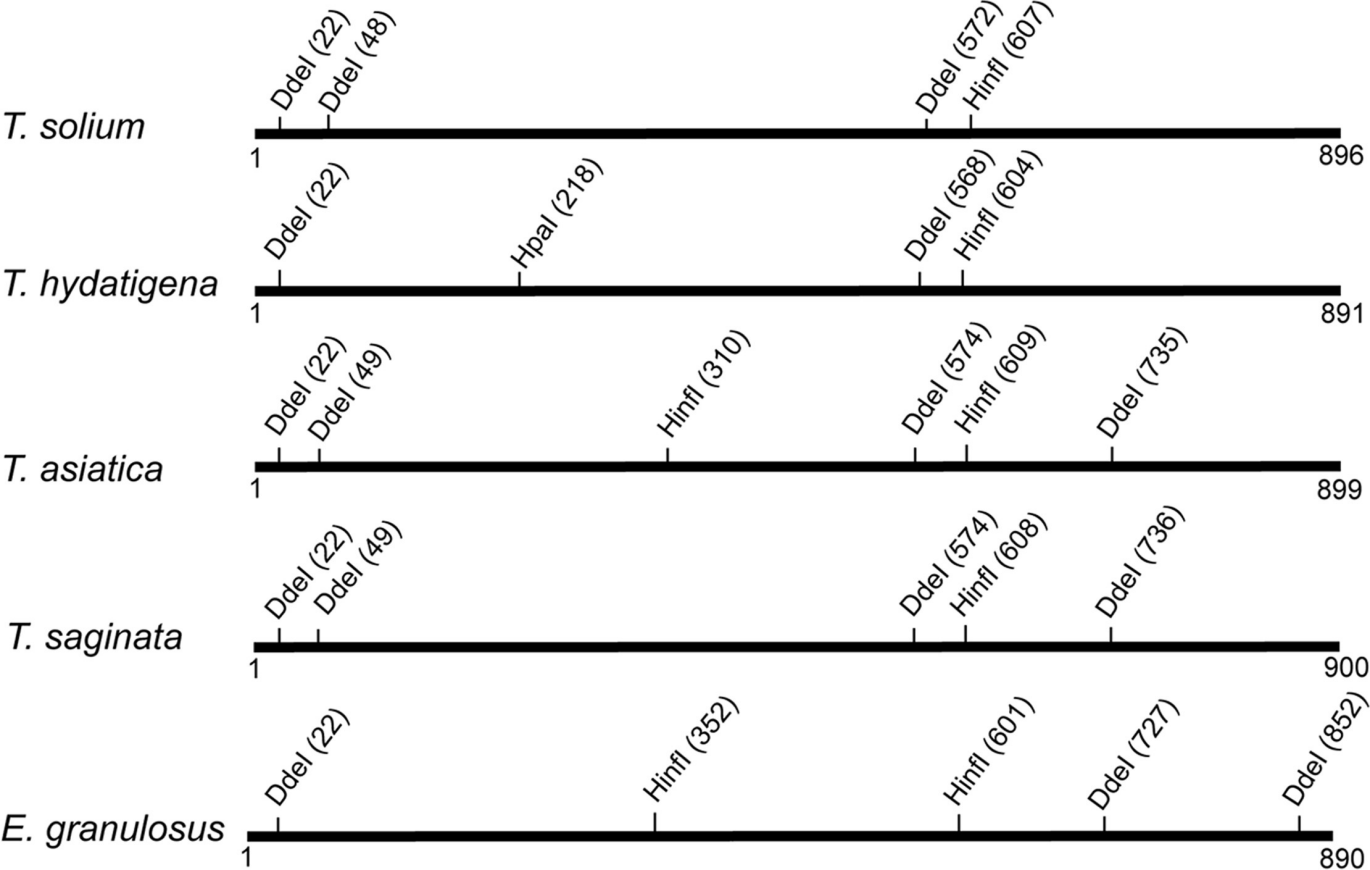
Schematic representation of predicted restriction enzyme sites occurring in 12S ribosomal DNA amplified from taeniid cestodes in PCR-RFLP. Numbers below each line represent the length of the 12S ribosomal DNA PCR product amplified from each species. Predicted restriction enzyme sites (DdeI, HinfI and HpaI) are shown above each gene product and the predicted position of enzyme sites for each species are shown in brackets.

To screen tissue samples and differentiate between *T*. *solium*, *Taenia hydatigena*, *Echinococcus granulosus* and *Taenia asiatica* infection (the most likely parasites to cause similar lesions to *T*. *solium*), PCR amplified products were digested using restriction enzymes to obtain fragments of the mitochondrial 12S ribosomal RNA gene. These fragments were separated by agarose gel electrophoresis to reveal distinct restriction fragment patterns for each taeniid species. The location of predicted restriction sites for the 12S ribosomal RNA gene for each of the taeniid species are shown in Fig 1.

Double digests of PCR products contained DdeI and HinfI in CutSmart buffer (New England Biolabs) in the same reaction tube, according to the manufacturer’s instructions and were prepared as also described by Somers et al. [14]. Single digests of PCR amplicons contained HpaI (New England Biolabs) in CutSmart buffer and were prepared as also described by Devleesschauwer et al. [15]. Restriction digested PCR products were separated by agarose gel electrophoresis (3% agarose in 45mM Tris-Borate, 1mM EDTA, pH 8.3). DNA restriction fragments separated by electrophoresis were stained with SYBR Green (Invitrogen) and visualized using a Safe Imager transilluminator (Invitrogen).

### DNA sequencing

The PCR products were separated by electrophoresis in 1.2% agarose (50mM Tris, 20mM sodium acetate, 2mM EDTA, pH 8.3), excised from the gel, purified using the Minelute purification kit (Qiagen) according to the manufacturer’s recommendations and quantified using a NanoDrop spectrophotometer. DNA sequencing was performed by Micromon (Melbourne, Australia) using the same primers as were used in the PCR reactions and a BigDye Terminator Cycle Sequencing kit (Applied Biosystems). PCR products were sequenced in both directions, assembled and analysed using Geneious 11.1 (Biomatters, Auckland, New Zealand). BLAST was used to compare the PCR-derived sequences with reference sequences of taeniid mitochondrial genomes in the GenBank database.

### Histology

Lesion specimens were recovered from pig tissues, trimmed to remove excess tissue and stored in 10% buffered formal saline. Paraffin embedded 5μm tissue sections were prepared and stained with haematoxylin and eosin.

## Results

### Lesions identified

**Fig 2:**
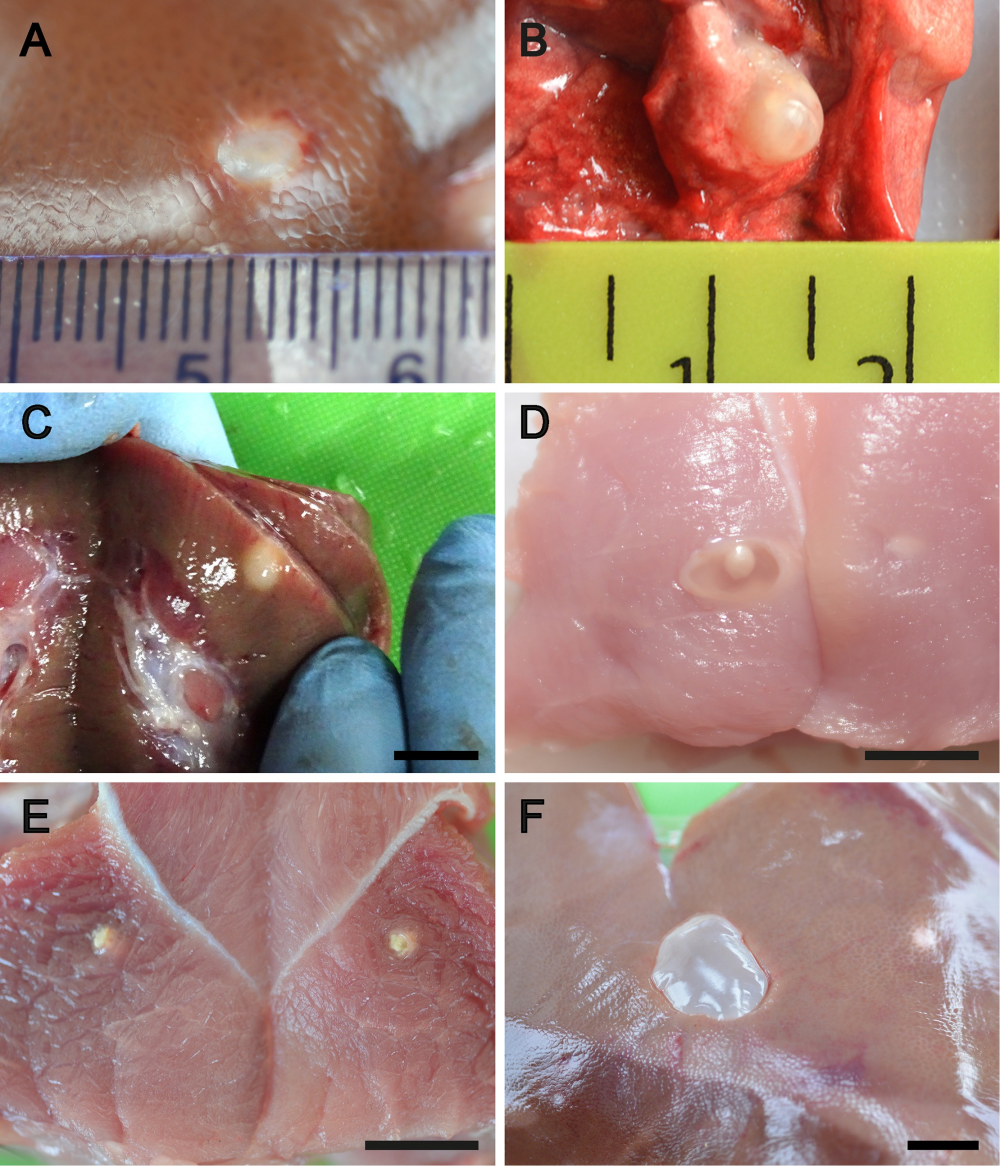
Examples of lesions detected in pigs from Uganda and Nepal that were either caused by *Taenia solium* or required further investigation to determine whether there was evidence that they were caused by *T*. *solium* or may have had had some other origin. A, B and C: Examples of lesions in the liver, lung and kidney for which no evidence could be found to determine their likely cause, scale small divisions in mm; D: Viable *T*. *solium* cysticercus in muscle tissue, scale approx. 1cm; E: non-viable lesion in muscle bisected during cutting of the tissue and inferred as being a non-viable *T*. *solium* cyst, scale approx. 1cm; F: mature, viable *Taenia hydatigena* cysticercus embedded in the surface of the liver, scale approx. 1cm.

**Fig 3:**
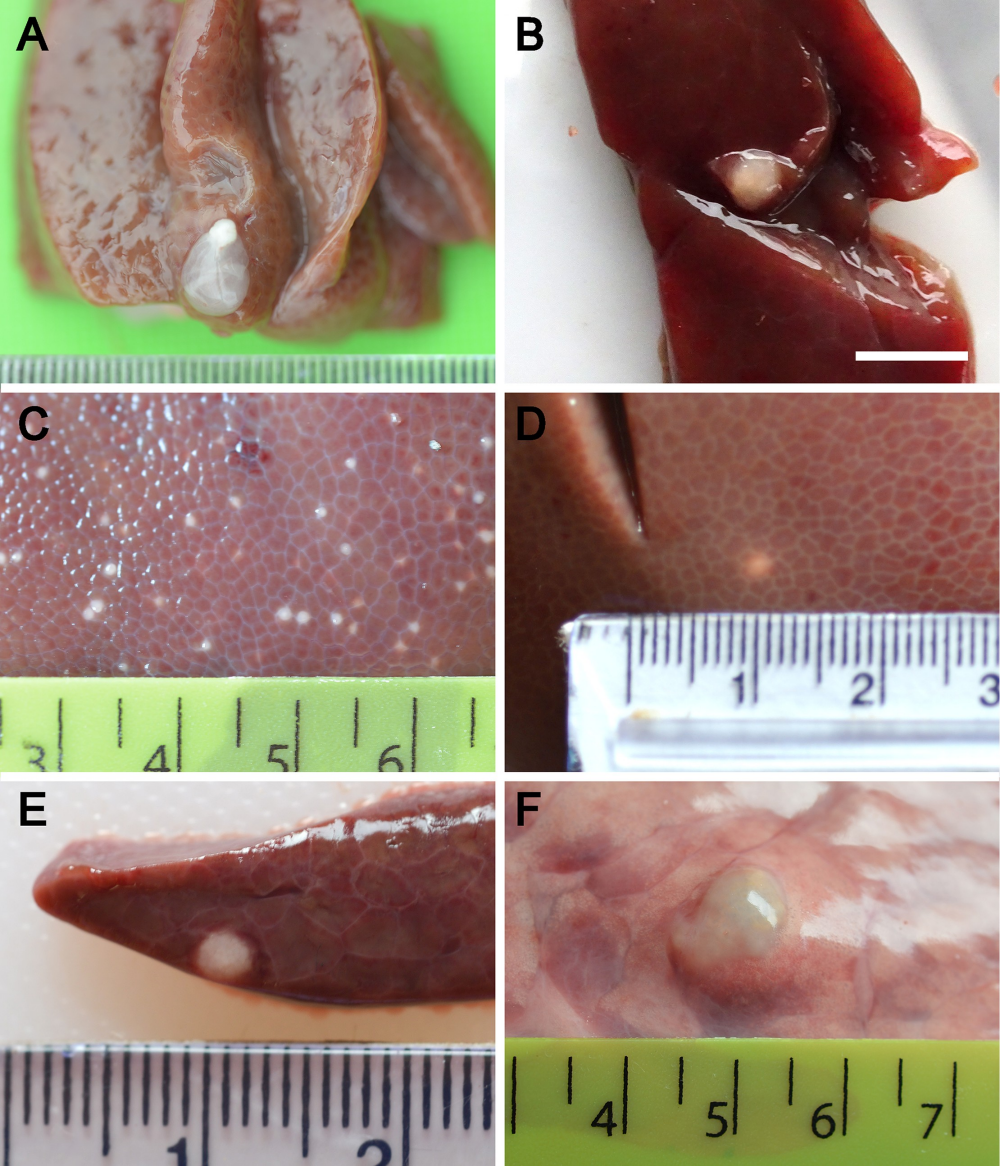
Further examples of lesions detected in pigs from Uganda and Nepal that were either caused by *Taenia solium* or required further investigation to determine whether there was evidence that they were caused by *T*. *solium* or may have had had some other origin. A: Immature, viable *T*. *hydatigena* cysticercus in the liver confirmed by DNA analyses, scale small divisions in mm; B: Non-viable lesion in the liver caused by *T*. *hydatigena* confirmed by DNA analyses, scale approx. 1cm; C, D: non-viable liver lesions in pigs from Nepal caused by *Taenia asiatica* confirmed by DNA analyses, scale large divisions in cm; E, F: Examples of smaller lesions cause by *Echinococcus granulosus* confirmed by DNA analyses in the liver and lungs of pigs from Nepal, scale large divisions in cm.

Lesions that could potentially be caused by *T*. *solium* were identified in the livers, lungs and kidneys, as well as in the tissue locations more commonly associated with *T*. *solium* (striated muscle and brain). Lesions were detected in the liver in 33% of the pigs from Uganda and 26% of the pigs from Nepal. Lesions were identified frequently in the lungs of animals in Nepal (35%) but less commonly in the animals from Uganda. Individual animals from both sites were also identified with lesions in the kidney and spleen. The lesions varied greatly in their macroscopic appearance (Figs 2 and 3) and included small (1-2mm) solid spots on the surface of the liver, with similar spots in the parenchyma of the liver or in the lung tissue, and larger, sometimes vesicular-looking lesions, in the liver and lung. Sometimes single lesions were present while at times there were several similar lesions or, occasionally, large numbers of similar lesions. The cause of the majority of lesions in the liver, lungs, spleen and kidney could not be determined by their macroscopic characteristics (Fig 2A, 2B and 2C). Lesions identified in the striated muscle were generally typical of viable or non-viable cysticerci of *T*. *solium* (Fig 2D and 2E respectively). Lesions that contained what was clearly identifiable as a cysticercus were also identified in the liver of some animals (Fig 2F, Fig 3A). In Nepal, but not Uganda, cysts were identified in both the liver and lungs that were clearly caused by *E*. *granulosus*, including cysts which contained germinal membrane, brood capsules and protoscolesces. In both Nepal and Uganda, mature *T*. *hydatigena* cysts were found in the liver, and were identified macroscopically.

**Fig 4:**
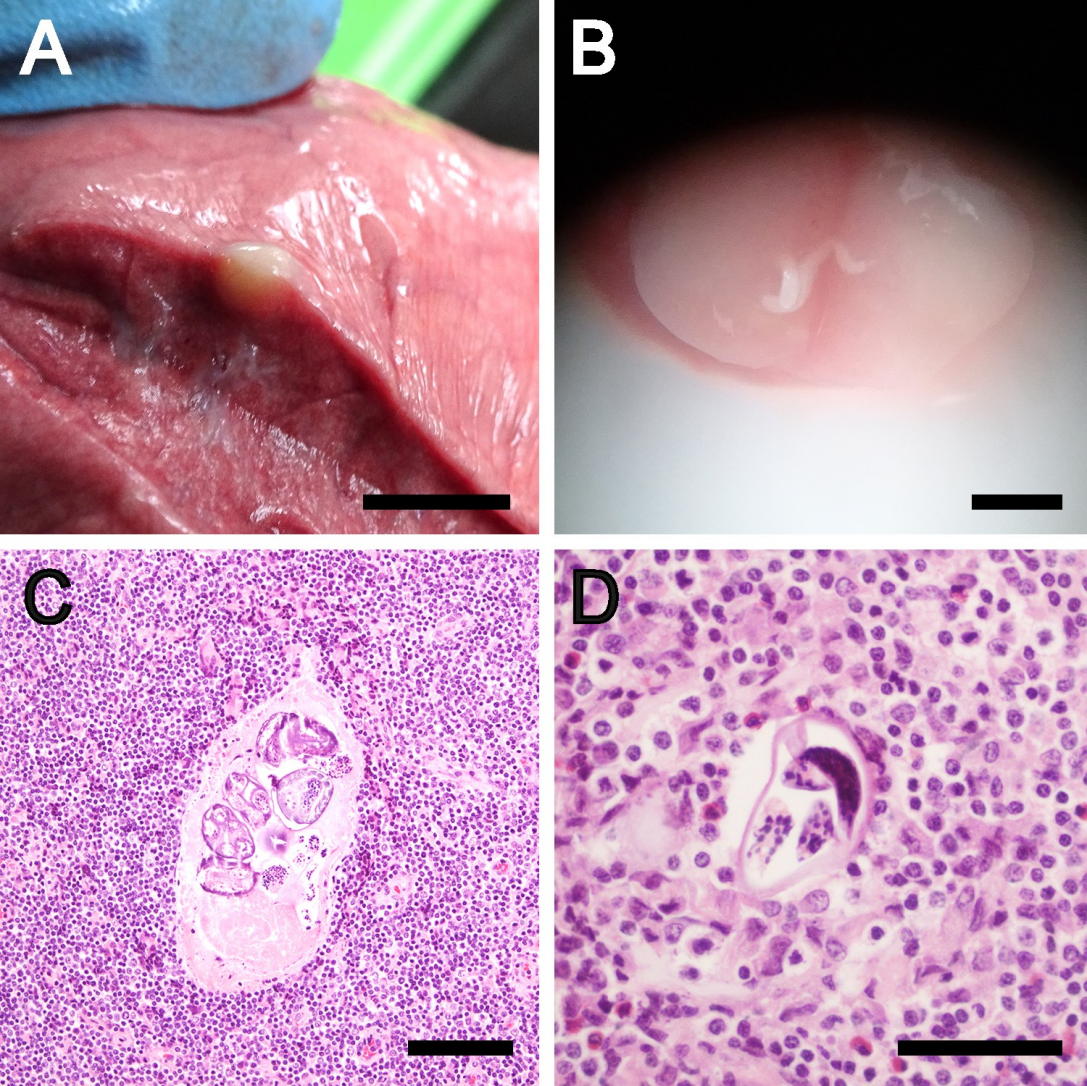
Lesion in the lung of a pig caused by *Metastrongylus spp*. A: macroscopic view of an uncut lesion, scale approx. 1cm; B: image taken through a dissection microscope of a cut lesion revealing the adult worm, scale approx. 1mm; C: histological section showing degenerate nematode cross-section with visible cuticle, musculature and uteri with embryos, scale 100μm; D: embryonated nematode egg, scale 50 μm.

On histological examination, lesions were often found to be foci of lymphoid hyperplasia due to antigenic stimulation, the cause of which was not apparent. Some had the appearance of migratory nematode tracts, with central necrosis surrounded by eosinophilic inflammation, although no definitive cause could be found. Some lesions in the lungs were found to have a filamentous inclusion within a solid mass (Fig 4A and 4B). On histological examination, a degenerate nematode was evident in cross-section with visible cuticle, musculature and uteri with embryos (Fig 4C) and/or containing embryonated nematode eggs approximately 50μm in size, consistent with *Metastrongylus* spp.

### DNA-based analyses of lesions found in Nepalese pigs

DNA was purified from representative examples of lesions found in pigs, particularly those found in tissues other than the striated muscle or brain, the origin of which could not be determined macroscopically. The samples were processed for PCR amplification using pan-nematode oligonucleotide primers however no amplification products were found, whereas amplification of control DNA from *Ascaris* spp. amplified an expected product of 166bp.

PCR amplification of the mitochondrial 12S ribosomal RNA gene using the TaenF and ITMTnR primers on control DNA samples resulted in PCR products of 890–900 bp for *T*. *solium*, *T*. *hydatigena*, *T*. *asiatica*, *T*. *saginata* and *E*. *granulosus*. For those DNA samples from tissue lesions which did amplify a product with the TaenF and ITMTnR primers, the products were within a comparable size range.

**Fig 5:**
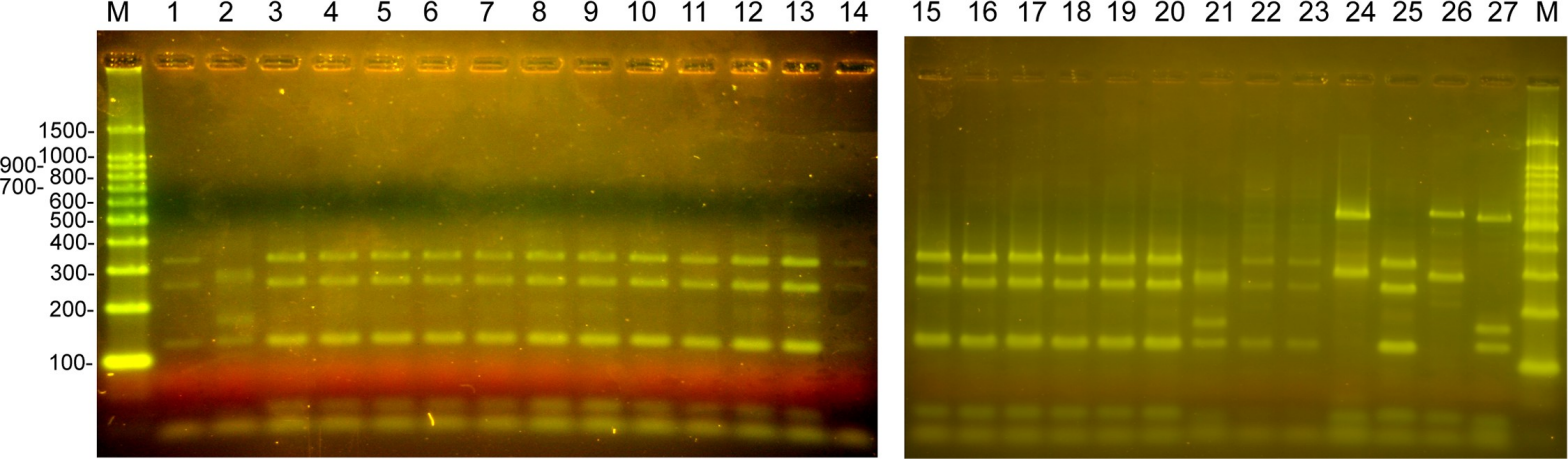
PCR-RFLP of DNA extracted from lesions recovered from pigs in Nepal. 3% agarose gels stained with SYBR green. Lanes 1–23, 12S PCR products digested with *Dde*I and *Hinf*I, derived from pig lesions. Lanes 24–27, 12S PCR products digested with *Dde*I and *Hinf*I from control DNA. (24) *T*. *solium*, (25) *E*. *granulosus*, (26) *T*. *hydatigena*, (27) *T*. *saginata*. (M) 100 bp DNA Ladder, Promega. For expected DNA fragment sizes refer to Fig 1.

Delineation of the 12S ribosomal RNA amplified products to species level was undertaken using RFLP (Fig 5) and DNA sequencing. Many of the Nepalese cystic lesions processed in RFLP following DdeI-HinfI digestion of the 12S amplicon produced a similar restriction digestion profile, consisting of four major DNA fragments (330 bp, 250 bp, 125 bp, 20–35 bp). This restriction profile was identical to the pattern obtained using control DNA purified from *E*. *granulosus* (Fig 5, lane 25) and was consistent with the predicted location of restriction sites (Fig 1). DNA sequencing of the 12S amplification product from putative *E*. *granulosus* cysts, identified in the Nepalese pig samples by PCR-RFLP, confirmed the parasite species as being *E*. *granulosus*.

**Fig 6:**
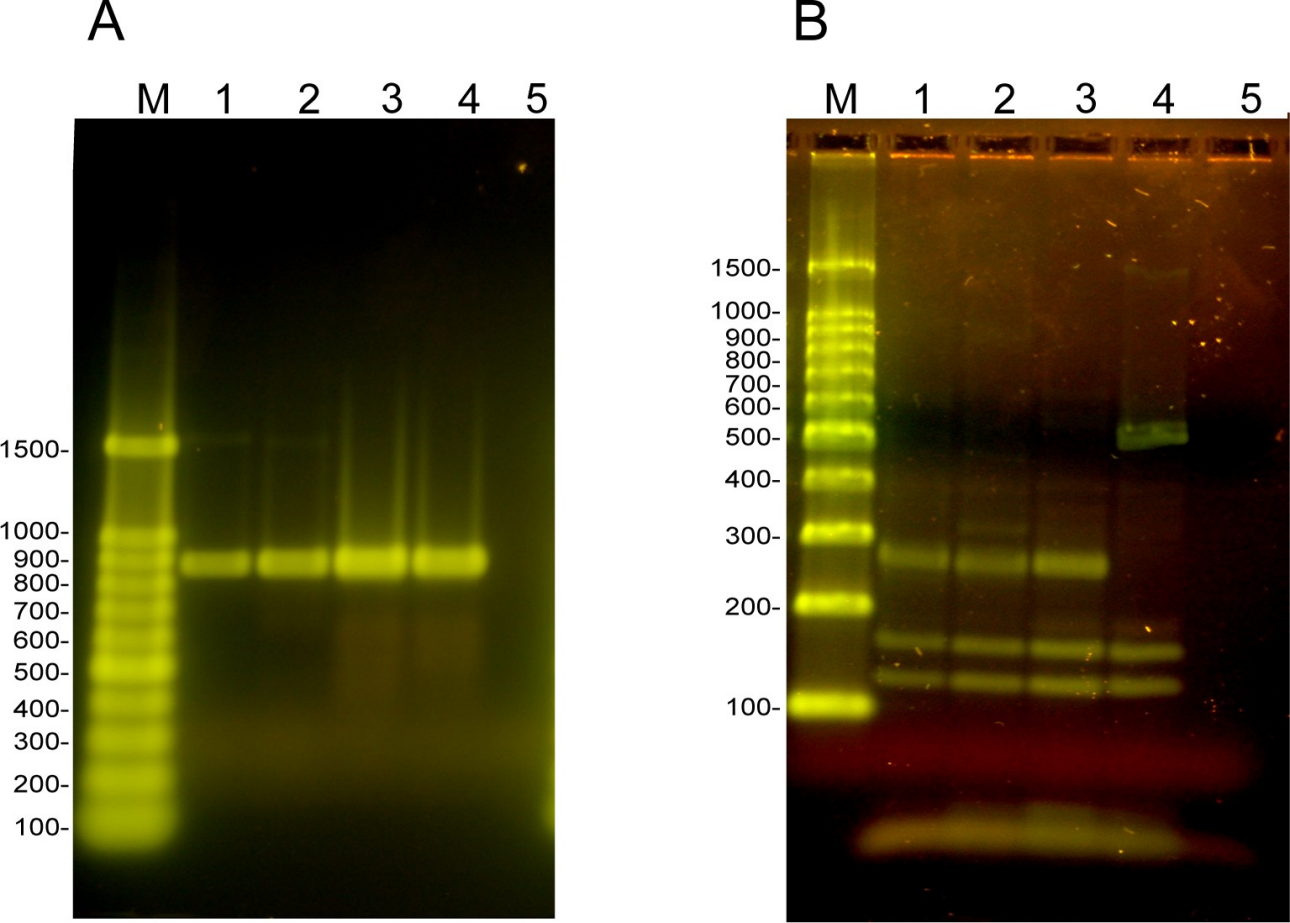
PCR-RFLP of DNA extracted from lesions confirming *T*. *asiatica* infection in Nepalese pigs. (A) 12S PCR products separated on 1.2% agarose gel stained with SYBR green. (B) 12S PCR products digested with *Dde*I and *Hinf*I and separated on 3% agarose gel stained with SYBR green. (1, 2) DNA from pig lesions, (3) *T*. *asiatica*, (4) *T*. *saginata*, (5) pig liver. (M) 100 bp DNA Ladder, Promega. For expected DNA fragment sizes refer to Fig 1.

DdeI-HinfI digestion of the PCR-RFLP DNA products obtained using DNA from lesions in the livers of two of the Nepalese pigs (Fig 3C and 3D; Fig 5, lanes 2 and 21) differed from the result obtained for the majority of lesions which were confirmed as being *E*. *granulosus* (Fig 5, lanes 3–20). These two PCR-RFLP patterns also differed from those obtained for the *T*. *solium*, *T*. *hydatigena* and *T*. *saginata* controls (Fig 5, lanes 24, 26, 27, respectively), but were identical to the pattern seen using control DNA from *T*. *asiatica* (Fig 6), indicating that these liver lesions were caused by *T*. *asiatica*. DNA sequencing of the 12S PCR product and BLAST comparisons confirmed that these two pigs had lesions caused by *T*. *asiatica*.

### DNA-based analyses of lesions found in Ugandan pigs

DNA was purified from representative examples of lesions found in pig tissues other than the striated muscle or brain, the cause of which could not be determined macroscopically. The samples were processed for PCR amplification using pan-nematode oligonucleotide primers, however no amplification products were found, whereas amplification of control DNA from *Ascaris* spp amplified an expected product of 166 bp.

**Fig 7:**
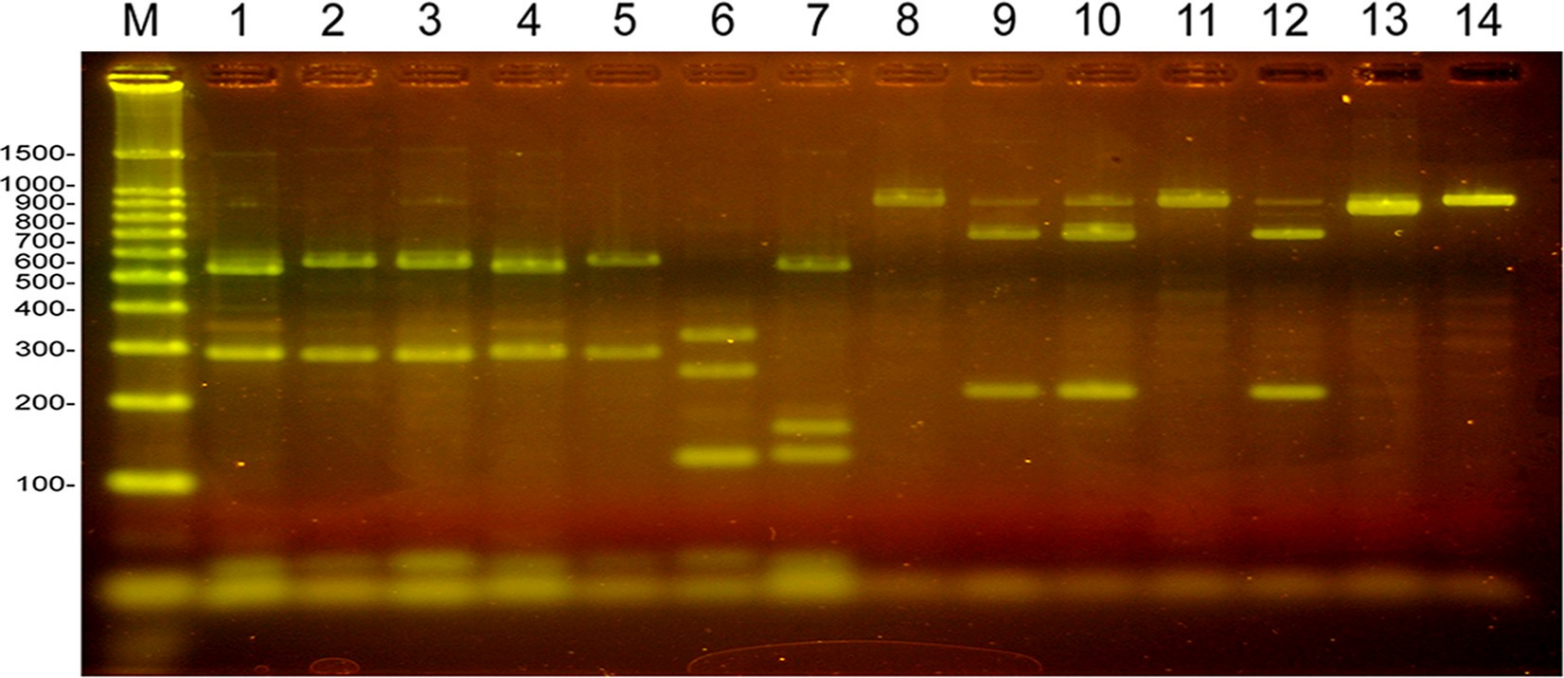
PCR-RFLP of DNA extracted from lesions recovered from pigs in Uganda. 3% agarose gel stained with SYBR green. Lanes 1–7, 12S PCR products digested with *Dde*I and *Hinf*I. Lanes 8–14, 12S PCR products digested with *Hpa*I. (1, 8), (2, 9) and (3, 10) are digested PCR products derived from the same lesion in the case of each pair. (4, 11) *T*. *solium*, (5, 12) *T*. *hydatigena*, (6, 13) *E*. *granulosus*, (7, 14) *T*. *saginata* control DNA. (M) 100 bp DNA Ladder, Promega. Lanes 1 and 8 are digested PCR products from a viable *T*. *solium* cysticercus from muscle tissue. For expected DNA fragment sizes refer to Fig 1.

For those DNA samples from tissue lesions which did produce a PCR amplification product of the mitochondrial 12S ribosomal RNA gene using the TaenF and ITMTnR primers, the products were within a comparable size range to those seen using control cestode DNA samples detailed above. RFLP analysis of the PCR product digested with DdeI-HinfI, followed by DNA sequencing in some cases, allowed the determination of the parasite species responsible for those lesions which did generate a 12S ribosomal RNA gene product in PCR. Samples from the livers of two pigs (Fig 3A and 3B) were unable to be clearly differentiated from being either *T*. *solium* or *T*. *hydatigena* following digestion with DdeI-HinfI in PCR-RFLP, since their restriction patterns (Fig 7, lanes 2 and 3), consisting of three DNA fragments (550 bp, 290 bp and 20–35 bp), were similar to both the *T*. *solium* and *T*. *hydatigena* controls (Fig 7, lanes 4 and 5 respectively). The restriction pattern of the two liver lesions obtained using DdeI-HinfI digestion, also differed significantly from the profile obtained for control DNA from *E*. *granulosus* and *T*. *saginata* (Fig 7, lane 6 and 7). Digestion of the 12S PCR products derived from these two lesions using HpaI (Fig 7, lanes 9 and 10) produced profiles consisting of two restriction fragments (670 bp, 220 bp) of the 890 bp PCR product, identical to the *T*. *hydatigena* control (Fig 7, lane 12). These patterns differed from those obtained with the *T*. *solium* control DNA (Fig 7, lanes 4 and 11) and from DNA obtained from a viable *T*. *solium* cyst from muscle (Fig 2D; Fig 7, lanes 1 and 8). DNA sequencing of the 12S PCR products from the two pig liver lesions shown in Fig 3A and 3B and BLAST comparisons confirmed they were caused by *T*. *hydatigena*.

## Discussion

In pigs from Nepal and Uganda, lesions were detected in body organs which are not normally sites of infection with *T*. *solium*, but which could potentially have been caused by *T*. *solium*. Although viable cysticerci were found in the liver of some animals, none were found to be cysticerci of *T*. *solium*. A wide variety of other lesions were also identified in various tissues, particularly in the liver and lungs. Approximately a third of the animals were found to have lesions in the liver which could, potentially, have been caused by *T*. *solium*, and, in Nepal, a quarter of the animals had lesions in the lungs which could have been confused with non-viable lesions caused by *T*. *solium*. The characteristics of the lesions varied greatly, both in their number in individual animals and in their macroscopic characteristics (Figs 2 and 3). The cause of many of the lesions was unable to be determined by macroscopic or microscopic observation or by histological or DNA investigations. However, the cause of some lesions was able to be determined definitively (Fig 2D–2F; Fig 3A–3F). Lesions were identified as having been caused by nematode infections, in both Uganda and Nepal by *T*. *hydatigena*, and in Nepal by *T*. *asiatica* and *E*. *granulosus*. No lesions in sites other than the striated muscles and brain were identified as having been caused by *T*. *solium*.

Several studies have detailed the tissue localization of *T*. *solium* cysticerci in naturally infected pigs which found the cysts to be restricted to the striated muscles and brain, as we did in this investigation. Boa et al. [8] undertook an extensive study involving detailed dissection of all the striated muscle tissue, brain and many body organs of 24 pigs from the Mbulu District of Tanzania with naturally-acquired *T*. *solium* infections, including 10 animals harbouring more than 10,000 cysts and one animal with >80,000 cysts. No *T*. *solium* cysts were found in any of the animals in the spleen, kidneys, lungs or liver. Phiri et al. [16] examined the tissue distribution of *T*. *solium* cysts in 31 naturally infected pigs from the southern and eastern regions of Zambia including the thorough slicing of body organs and found no cysts in the spleen, kidneys, lungs or liver. Similarly, Singh et al. [9] undertook thorough slicing of body organs of 19 naturally infected pigs from the Punjab region of India and found no cysts in the spleen, kidneys, lungs or liver. Onah and Chiejina [17] examined 483 naturally infected pigs from Enugu State in Nigeria by extended meat inspection, although they did not undertake tissue slicing. Among the 483 infected animals examined, 403 animals were found to have what was referred to as generalized infections, most with ‘vast numbers’ of cysticerci. They comment that “despite the heavy infections encountered, no cysts were found in the kidneys, liver or lungs of any pigs”.

Infections with *T*. *solium* cysticerci have been described in organs other than the striated muscles or brain in animals experimentally infected with *T*. *solium* from China [18–20]. Small numbers of *T*. *solium* cysts have been reported in a small number of naturally infected young piglets from Mexico [21] and two cysticerci were identified in the liver of mature pigs from Nepal [22], however neither study included clear evidence to differentiate these as being *T*. *solium* rather than either *T*. *hydatigena* or *T*. *asiatica*. In his treatise on meat inspection, Ostertag [6] comments that while *T*. *solium* cysts are normally found in the muscles, they may at times be seen in the lymph nodes and subcutaneous fat, while in an even more extensive infection the parasites may be present also in the liver and the lungs.

In addition to these poorly substantiated reports of the occasional occurrence of *T*. *solium* cysts occurring in pigs in tissue locations other than the striated muscles and brain, one report stands out as the only instance where cysts were commonly found in naturally infected pigs in other body organs. Chembensofu et al. [7] undertook a detailed study of the tissue distribution of *T*. *solium* cysts in 37 naturally infected pigs from Katete and Sinda Districts in the Eastern Province of Zambia. Ten animals (27%) were diagnosed as having cysts in the liver, one with a cyst in the spleen and two with cysts in the lungs. At least one cyst from every organ found to be infected was confirmed as being *T*. *solium* by PCR-RFLP, which would appear to provide definitive evidence that they were indeed *T*. *solium*.

Chembensofu et al. [7] did not provide supportive information such as figures to aid in the description of the characteristics of cysts found in unusual locations, or the PCR-RFLP results. No information was given about what controls were used in the PCR-RFLP analyses, especially controls that would indicate contamination was unlikely to have been possible at the time the specimens were collected. To the best of our knowledge, we used the same PCR-RFLP methods as those used by Chembensofu et al. [7], although detailed methods were not provided directly by Chembensofu and colleagues, nor were they included in the cited references. When tissues of a pig that has a heavy infection with *T*. *solium* cysts are being sliced by hand, the instruments and cutting boards etc become extensively contaminated with whole cysticerci, bladder fluid, cyst walls and scolesces. Meticulous care would be required to ensure that there was no possibility that specimens collected from one animal could not be contaminated with DNA from a previously dissected animal or from cysts from other tissues of the same animal. In our study, particular care was taken in this regard. Specimens for DNA analyses also included tissue samples from the same organ adjacent to the area where a lesion was excised but containing no lesion. None of these control tissue samples amplified a PCR product indicative of the presence of DNA from a cestode parasite.

We attempted to use “pan-nematode” primers [11] in PCR in order to screen DNA from lesions found in the liver or lungs of pigs that had the potential to be interpreted as being caused by *T*. *solium*, however none of the samples generated a PCR product. Clear evidence was obtained from lung lesions as being associated with nematode parasites (Fig 4), likely to be *Metastrongylus* spp. While the PCR primers developed by Poon et al. [11] are referred to by them and us as pan-nematode, the primers were not designed with the COX1 sequence from *Metastrongylus* spp. Our subsequent characterisation of the *cox1* gene sequences corresponding to the primer locations in *Metastrongylus* spp found substantial differences in the downstream primer sequence in *Metastrongylus*, providing an explanation for the lack of amplification of a PCR product in those instances.

We found that PCR-RFLP using primers derived from the mitochondrial 12S ribosomal RNA gene was a valuable tool for species identification of *Taenia* and *Echinococcus* and for pre-screening many samples prior to DNA sequencing. Our use of “pan-cestode” PCR primers [23] annealing to a conserved region of the 12S ribosomal RNA gene, allowed the amplification of a fragment of the gene from *T*. *solium*, *T*. *hydatigena*, *T*. *asiatica*, *T*. *saginata* and *E*. *granulosus*. For those samples where a PCR product was amplified, the methodology used definitively differentiated suspect lesions as being caused by a particular species of *Taenia* or *Echinococcus*.

Ooi et al. [24] list eight helminth parasites that produce lesions in the liver of pigs other than *T*. *solium*. These include *Schistosoma japonicum*, *Taenia hydatigena*, *T*. *asiatica*, *E*. *granulosus*, *Echinococcus multilocularis*, *Ascaris suum*, *Toxocara canis* and *Stephanurus dentatus*. We confirmed the presence of viable and non-viable lesions caused by *T*. *hydatigena* (eg. Fig 3A and 3B) among the liver specimens examined here, and also two animals from Nepal with *T*. *asiatica* infection (Fig 3C and 3D; Fig 6B). The two animals infected with *T*. *asiatica* were derived from different village locations in the Banke District, one from Khatikanpurwa and one from Mahapurwa. A previous study reported *T*. *asiatica* infecting pigs in Nepal [25]. Our investigations to determine whether the *T*. *asiatica* lesions had genetic features consistent with being *T*. *asiatica* or being hybrids of *T*. *asiatica* and *T*. *saginata* [26] were inconclusive using criteria according to Sato et al. [27].

Hepatic lesions were found in the livers of animals in both Uganda and Nepal which contained a viable cysticercus. In most cases the cysticercus had characteristics typical of *T*. *hydatigena* (Fig 2F). However, one instance occurred when a cysticercus was found that was of a size more typical of *T*. *solium* (Fig 3A). The scolex of this cysticercus contained two rows of hooks, the largest of which had a mean length of 170 μm, while the mean length of the small hooks was 113 μm. Speciation of this cysticercus based only on hook length was not possible since the hooks were within a size range for both *T*. *hydatigena* and *T*. *solium* [28]. PCR-RFLP and sequencing of DNA from this cyst confirmed it as being an immature *T*. *hydatigena* cysticercus.

Among the 167 pigs which were examined in this study, six animals were identified which had only a single caseous or calcified lesion in the muscle tissue, the cause of which could not be definitively identified. As there are numerous potential causes of caseous or calcified lesions in muscle tissue other than *T*. *solium*, we applied the same diagnostic criteria as was applied by Sah et al. [22] and Poudel et al. [10] in not classifying these animals as *T*. *solium* infection. Animals found to have two or more caseous or calcified lesions in the muscle tissue were classified as being cases of *T*. *solium* infection since this is the most likely cause of multiple non-viable lesions in the muscle tissues of pigs reared under free-roaming conditions in countries that are endemic for *T*. *solium*.

We did not find any tissue lesion, other than in the striated muscles and brain, in free-ranging pigs from Uganda and Nepal which could be proven to be caused by *T*. *solium*. These findings are consistent with those of all previous studies of the tissue localization of cysticerci in naturally infected pigs where meticulous dissections of mature animals has been undertaken, with the exception of the study recently published by Chembensofu et al. [7]. Their re-visiting of necropsy for detection of porcine cysticercosis in 38 infected pigs from Zambia found 27% of the animals to have *T*. *solium* cysticerci in the liver, as well as some animals having *T*. *solium* in the lungs and spleen. *T*. *solium* may occur sporadically in aberrant tissue locations, as has been mentioned anecdotally by others, for example by Osterag [6]. However, it is difficult to reconcile one study finding as many as 27% of pigs being infected with *T*. *solium* in the liver, when no animal was found to have *T*. *solium* in the liver among a larger total number of animals investigated by others [8, 16; this study], including pigs from the same Zambian Province [16] from which Chembensofu et al. [7] sourced some of their animals.

Future studies involving comprehensive evaluation of porcine cysticercosis by necropsy, that may include investigation of organs such as the liver, lungs and spleen, should take appropriate measures before concluding that lesions found in aberrant locations were caused by *T*. *solium*. Any DNA analyses undertaken on lesions in aberrant tissue locations that are suspected to have been possibly caused by *T*. *solium* should be supported by evidence from appropriate control samples. Clearly enunciated steps should also be taken to minimize the possibility of contamination of necropsy specimens with *T*. *solium* DNA from a different animal or tissue location.
